# Development and Feasibility Testing of a Multilevel Intervention to Increase Hepatitis C Virus Screening Among Baby Boomers in Primary Care

**DOI:** 10.1007/s13187-023-02268-x

**Published:** 2023-02-17

**Authors:** Monica L. Kasting, Alfu Laily, Lauren D. Nephew, Cleveland G. Shields, Rivienne Shedd-Steele, Susan M. Rawl

**Affiliations:** 1 Department of Public Health, Purdue University, 812 W. State Street, Room 216, West Lafayette, IN 47907 USA; 2 Cancer Prevention and Control Program, Indiana University Simon Comprehensive Cancer Center, Indianapolis, IN USA; 3 Department of Medicine, Division of Gastroenterology and Hepatology, Indiana University School of Medicine, Indianapolis, IN USA; 4 Department of Human Development and Family Sciences, Purdue University, West Lafayette, IN USA; 5 Indiana University School of Nursing, Indianapolis, IN USA

**Keywords:** Hepatitis C, Primary health care, Intervention development, Multilevel intervention, Screening, Health services research

## Abstract

Chronic infection with hepatitis C virus (HCV) results in an increased risk of cirrhosis and hepatocellular carcinoma (HCC). Only 15% of baby boomers (born 1945–1965) have ever been screened. We aimed to develop a multilevel intervention to increase HCV screening for baby boomers in a primary care setting. This study included two phases: intervention development (*phase 1*) and feasibility testing (*phase 2*). In phase 1, we partnered with a Community Advisory Board and a Provider Advisory Board to develop a multilevel intervention to increase HCV screening to be delivered to both providers and patients in primary care. Phase 2 assessed intervention feasibility, acceptability, and usability by conducting Concurrent Think Aloud (CTA) interviews and surveys using previously validated scales with patients (*n* = 8) and providers (*n* = 7). *Phase 1 results:* The patient-level intervention included a mailed reminder letter and CDC pamphlet and a 7-min in-clinic educational video. The provider-level intervention included a 30-min educational session and monthly performance feedback e-mails. *Phase 2 results:* Qualitatively, both the patient and provider-level intervention were feasible, acceptable, and usable by the target audiences. Quantitatively, on a 1–4 scale, the range of patient-level scores was 3.00–4.00 and provider level was 3.50–4.00 for feasibility, acceptability, and usability. This intervention could improve HCV screening among a high-risk population and therefore reduce HCV-related morbidity and mortality. This project developed a feasible, acceptable, and usable multilevel intervention aimed at increasing HCV screening in primary care.

## Introduction

Hepatitis C virus (HCV) infects approximately 41,000 people in the USA each year, and more than half of those develop a chronic infection [1]. Half of all liver cancer cases in the USA are caused by chronic HCV infections [1]. HCV infection is unequally distributed in the population; 75% of persons with chronic HCV were born between 1945 and 1965 (baby boomers) [1]. The US Department of Health and Human Services Viral Hepatitis National Strategic Plan has a goal to increase the proportion of people who are tested and aware of their viral hepatitis status [2]. The US Preventive Services Task Force (USPSTF) has recommended one-time universal screening for all baby boomers since 2013 and in March, 2020, they updated their recommendation to include all people aged 18–79 years [3]. Yet, self-report data from the National Health Interview Survey as well as electronic medical record (EMR) data collected from 2015 to 2017 showed that only 12–14% of baby boomers have ever been screened for HCV [4, 5]. Almost half of people infected with HCV do not recall or report having specific risk factors [6]. Thus, risk-based screening fails to identify almost half of people chronically infected with HCV, and universal screening will identify those missed by risk-based screening alone.

Previous research identified multiple barriers to HCV screening including lack of time during a clinic visit and competing priorities that supersede HCV screening [7]. Some interventions have focused on increasing HCV screening, but most targeted only one level or only marginally increased screening [8–10]. Therefore, this study aimed to (1) develop a multilevel intervention directed at primary care patients and providers to increase HCV screening rates among baby boomers and (2) determine baby boomer patients’ and providers’ perceptions of intervention feasibility, acceptability, and usability.

## Methods

This study consisted of two phases: intervention development (phase 1) and feasibility testing (phase 2). We developed a multilevel intervention prototype aimed at patients and providers to address barriers to HCV screening [7] based on our work, previous literature, theory-based interventions, and barriers and facilitators to HCV screening specifically [9, 10]. This study was reviewed and approved by the authors’ Institutional Review Board.

### Phase 1: Intervention Development

#### Prototype Development

The patient-level prototype consisted of a reminder letter mailed prior to a clinic visit, an informational pamphlet from the Centers for Disease Control and Prevention (CDC), and a brief (< 10 min) tablet-based in-clinic educational session once the patient arrives for their appointment. Content was informed by the competing demands model (CDM) [7] and health belief model (HBM) [11] and was designed to educate primary care patients about HCV infection, transmission, insurance coverage/cost, HCV-infection susceptibility, HCV severity, and screening benefits.

The provider-level intervention prototype included a one-time, educational session (< 30 min), designed to be in-person but could be converted to virtual, if necessary. It was followed by monthly performance feedback e-mails, to highlight the providers’ screening rate over time. Information included in the provider educational session was effective communication with patients about HCV, barriers [7], linkage to care [12], and needs for patients who screen positive [12].

Development of the patient-level intervention was guided by a Community Advisory Board (CAB), and the provider-level intervention was guided by a Provider Advisory Board (PAB) to engaging relevant members of the target users in the intervention development process [13]. We had three separate meetings with both the CAB and the PAB over a 6-month period from September 2020 to March 2021, and the intervention was developed iteratively with revisions made after each meeting.

#### Community Advisory Board

We partnered with the Office of Community Outreach and Engagement at the Indiana University Simon Comprehensive Cancer Center to recruit CAB members. The CAB consisted of four baby boomer members, all born 1945–1965, who were racially and ethnically diverse to reflect the overall clinic population. The median age was 65 years (range: 64–74), 3 of the 4 were female, 2 were Black, 1 was White, and 1 was Hispanic. The CAB provided feedback on intervention design, content, length, messages, graphics, and preferences for delivery.

#### Provider Advisory Board

The PAB was recruited through professional contacts of the study team. The PAB consisted of 2 primary care providers and 2 gastroenterologists, 3 of the 4 members were female; 1 was Black, 1 was Hispanic, and 2 were White. Each PAB member participated in three meetings. During these meetings, PAB members provided feedback on design, content, length, messages, graphics, preferences for delivery, and ways to facilitate engagement. In addition, while providers did not provide feedback on the patient-level intervention, one PAB member did review the patient-level intervention to ensure accuracy.

### Phase 2: Feasibility, Acceptability, and Usability Testing

We evaluated feasibility, acceptability, and usability of the intervention with 8 patients and 7 providers. Community members who were born 1945–1965, able to read and speak English, had access to a computer, and had not served on our CAB were eligible to participate. Providers were eligible if they were practicing family medicine or internal medicine physicians, delivered care to patients born 1945–1965, able to read and speak English, and were not members of our PAB. Interviews lasted approximately 1 h and used concurrent think aloud (CTA) [14] techniques to assess intervention reactions, comprehension, and perceptions of the strengths and areas that could be improved.

#### Patient-Level Recruitment

Community members were recruited using the All IN for Health *Trial*_*X*_* iConnect*, a HIPAA-compliant, secure, public-facing research recruitment platform provided by the Indiana Clinical and Translational Sciences Institute (CTSI). Interested participants contacted the study team and were provided study information, confirmed interest and eligibility, obtained informed consent, and scheduled the 1-h interview.

#### Provider-Level Recruitment

Provider recruitment was facilitated by the Indiana CTSI Office of Strategic Operations, which sent an initial e-mail to eligible providers. Interested providers then contacted the study team via e-mail and completed a brief phone call to provide additional study information, confirm interest and eligibility, obtain informed consent, and schedule the one-hour interview.

#### Data Collection

##### Qualitative Interviews

All interviews were conducted virtually in June 2021, and the screen-sharing feature was used to show intervention content. Patient interviews had a median length of 51 min (range: 45–62 min); provider interviews had a median length of 49 min (range: 45–59 min). Community members and providers viewed their respective intervention components and provided their reaction, understanding of the materials, and strengths and limitations of the intervention. Interviews were audio-recorded, transcribed verbatim, coded, and analyzed.

##### Quantitative Survey

Following the interviews, patients and providers completed a brief (5-min) quantitative survey. The surveys were distinct for the two groups, but both used previously validated scales where possible and assessed feasibility [15–18], acceptability [15–17], usability [15, 17, 18], user-friendliness [17, 18], credibility [17, 18], comprehensibility [17, 18], and readability [16–18]. Following completion of the survey, participants were redirected to a separate survey to enter their information to receive a gift card.

#### Data Analysis

##### Qualitative

Transcribed interviews were analyzed by two authors trained in qualitative data analysis (MLK and AL) using methods described by Cooke which involves coding quotes into categories developed a priori [19]. These categories included participants’ observation of the intervention, interpretation of the information, and comments regarding feasibility, acceptability, and usability.

##### Quantitative

The survey assessed each intervention item separately and included questions on a 4-point Likert scale from strongly agree to strongly disagree. Items were scored so that higher values indicated higher feasibility, acceptability, and usability. The intervention was considered feasible, acceptable, and usable if the median score was ≥ 3 out of 4 on the items measuring each construct. Survey responses were analyzed using medians and ranges.

## Results

### Phase 1

The final patient-level intervention (see Table 1 for content and visuals) included a reminder letter and CDC informational pamphlet [20] mailed to the patients’ home prior to an upcoming primary care appointment, followed by a 7-min-long in-clinic educational video intended to be viewed in the waiting room during their clinic visit. The reminder letter included information about their upcoming appointment, informed them they are due for HCV screening, and gave brief information about HCV infection. The CDC pamphlet augmented the letter with more detailed information about HCV. The video included general information on HCV infection, transmission, symptoms, screening, treatment, and resources for low or no cost screening and treatment. It concluded with messages to activate the patient to talk to their doctor about being screened at their appointment that day.

**Table 1:**
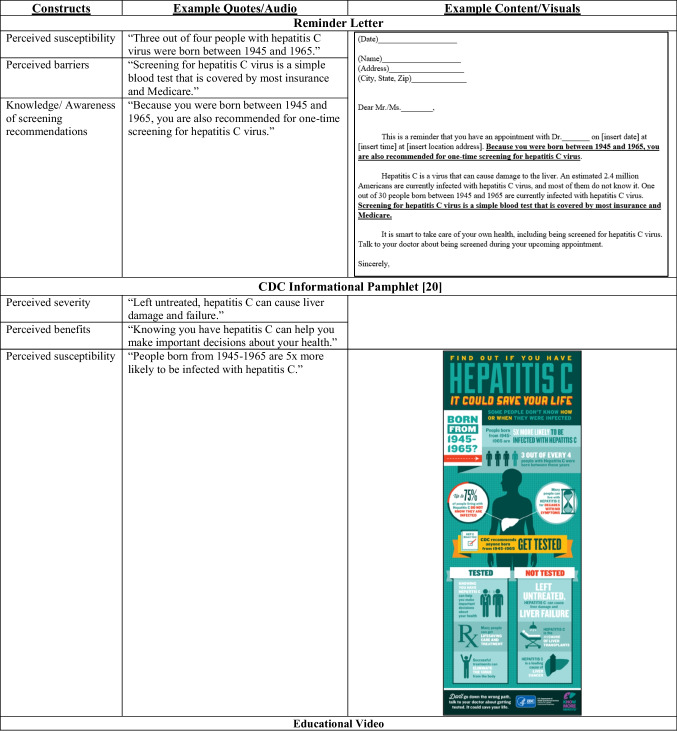

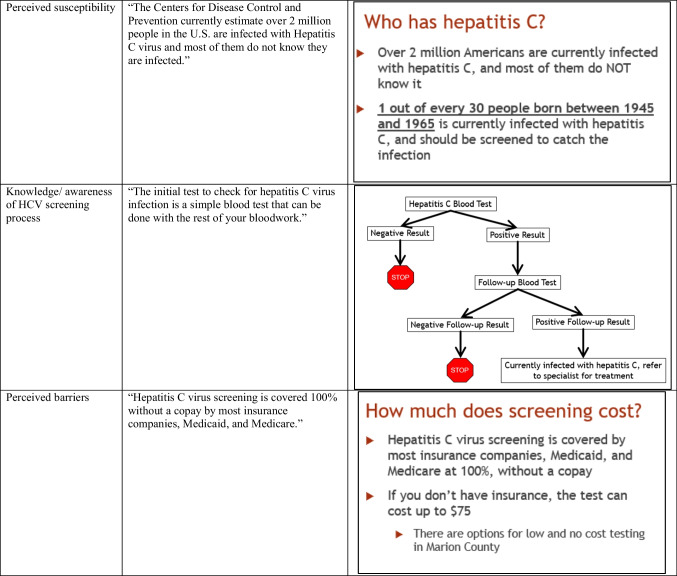
Patient intervention content

The final version of the provider-level intervention (see Table 2 for content and visuals) included a 30-min educational session followed by monthly performance feedback e-mails. The educational session included information on HCV epidemiology, natural history, screening recommendations, screening rates, screening steps, and a description of the primary care provider’s role compared to gastroenterologists’ role. The session ends by addressing common barriers and provides resources to address these barriers. The performance feedback e-mails then gave providers information on their HCV screening of eligible patients over time.

**Table 2:**
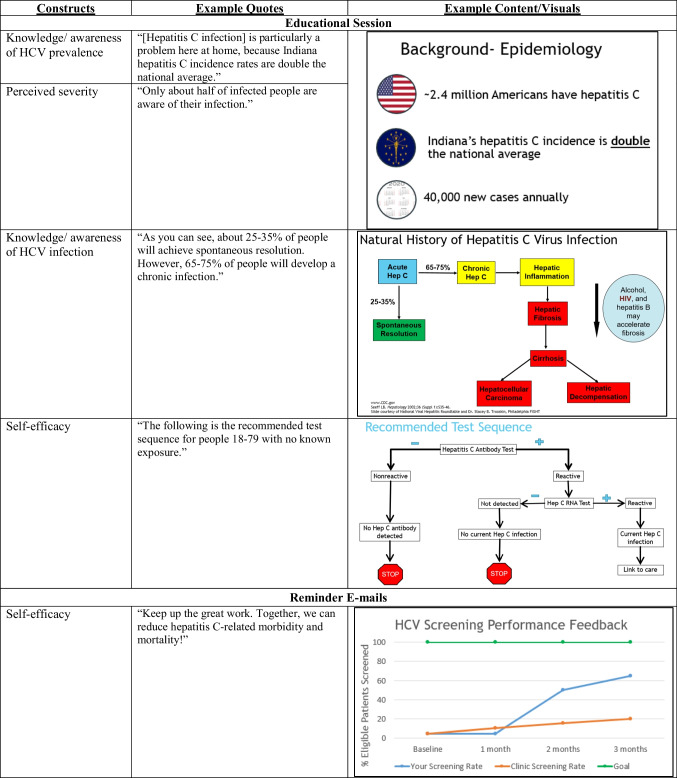
Provider intervention content

### Phase 2

#### Participants

The patient sample (*n* = 8) consisted of 4 males and 4 females. Median age was 66.5 years (range: 58–74). Two participants were Hispanic, 3 were non-Hispanic White, 1 was non-Hispanic Black, and 2 were non-Hispanic biracial. The provider sample consisted of 7 physicians (5 family medicine, 2 internal medicine). They reported a median of 11.0 (range: 4.0–33.0) years in practice. Median age was 41.0 years (range: 34.0–65.0), and the majority (*n* = 5) were female. One was Hispanic, 4 were non-Hispanic White, 1 was non-Hispanic Black, and 1 was non-Hispanic biracial.

#### Patient Intervention

In the survey, each part of the intervention (reminder letter, CDC pamphlet, and educational video) had a median score of at least 3 out of 4 on all constructs measured (Table 3). While some scores on individual items did fall below 3, the median scores for the constructs were all at least 3.

**Table 3:** Patient-level intervention items and construct scores

REMINDER LETTER		
Construct (Cronbach’s alpha for reliability)	Items	Median score (range)
Acceptability (0.655)	The information in the letter will help me stay healthy	3.00 (3.0–4.0)
	The information in the letter was relevant to me	3.00 (2.0–4.0)
	I was satisfied with the information included in the letter	3.00 (2.0–4.0)
	I don’t really need the information in the letter	3.00 (1.0–4.0)
	This letter is a waste of time	4.00 (3.0–4.0)
	*Total acceptability median score*	*3.30 (2.4*–*3.8)*
Comprehensibility (0.533)	The information in the letter was easy to understand	3.00 (2.0–4.0)
	The letter helped me to understand the information about hepatitis C	3.50 (3.0–4.0)
	*Total Comprehensibility median score*	*3.50 (2.5*–*4.0)*
Feasibility (0.842)	The information in the letter was relevant	4.00 (2.0–4.0)
	The information in the letter was useful	3.50 (3.0–4.0)
	A letter like this could help me talk to my doctor about hepatitis C screening	4.00 (3.0–4.0)
	I would open the letter if it came in the mail	3.50 (3.0–4.0)
	I would pay attention to the information in the letter	3.50 (3.0–4.0)
	*Total feasibility median score*	*3.70 (2.8*–*4.0)*
Readability (N/A)	I liked the way the reminder letter looked	3.00 (3.0–4.0)
	The letter was easy to read	3.00 (3.0–4.0)
	*Total readability median score*	*3.00 (3.0*–*4.0)*
CDC PAMPHLET		
Construct	Items	Median score (range)
Acceptability (0.829)	The information in the CDC pamphlet was useful	3.50 (2.0–4.0)
	The information in the CDC pamphlet is relevant to me	3.00 (2.0–4.0)
	I really don’t need the information in the CDC pamphlet	3.50 (3.0–4.0)
	This CDC pamphlet is a waste of time	4.00 (2.0–4.0)
	I will tell other people about the information I learned in the CDC pamphlet	3.00 (1.0–4.0)
	*Total acceptability median score*	*3.40 (2.2*–*4.0)*
Comprehensibility (N/A)	The information in the CDC pamphlet was easy to understand	3.00 (3.0–4.0)
	The information in the CDC pamphlet will help me stay healthy	2.50 (2.0–3.0)
	*Total comprehensibility median score*	*3.00 (2.5*–*3.5)*
Credibility (N/A)	I trust the information presented in the CDC pamphlet	3.50 (2.0–4.0)
Readability (N/A)	I liked the way the CDC pamphlet looked	3.00 (2.0–3.0)
	The words in the CDC pamphlet were easy to read	3.00 (3.0–4.0)
	*Total readability median score*	*3.00 (3.0–3.0)*
EDUCATIONAL VIDEO		
Construct	Items	Median score (range)
Acceptability (0.911)	I was satisfied with the information presented	3.00 (3.0–4.0)
	I wanted more information from the video	3.00 (2.0–4.0)
	The video gave too much information	3.50 (2.0–4.0)
	The video is waste of time	4.00 (3.0–4.0)
	The information in the video was interesting	3.00 (3.0–4.0)
	The information in the video was relevant to me	3.00 (2.0–4.0)
	The information in the video was important to me	3.00 (2.0–4.0)
	The information was useful	3.00 (3.0–4.0)
	I can use the information from the video in my daily life	3.00 (2.0–4.0)
	I would like to learn more about hepatitis C screening	3.00 (2.0–4.0)
	I am going to ask my doctor about getting screened for hepatitis C	3.50 (3.0–4.0)
	I would recommend this video to my friends and family	3.00 (3.0–4.0)
	The presenter’s voice was pleasant	3.00 (3.0–4.0)
	The pace of the video was too slow	3.00 (2.0–4.0)
	The pace of the video was too fast	3.00 (3.0–4.0)
	*Total acceptability median score*	*3.10 (2.8*–*3.9)*
Comprehensibility (0.842)	Following the video was easy	4.00 (3.0–4.0)
	The information in the video was in a logical order	3.50 (3.0–4.0)
	The information was easy to understand	3.50 (3.0–4.0)
	The information will help me stay healthy	3.00 (3.0–4.0)
	I know where to find helpful resources for hepatitis C screening	3.00 (2.0–4.0)
	I learned something new about hepatitis C screening from the video	3.50 (2.0–4.0)
	I changed my opinion about hepatitis C because of this video	3.00 (2.0–4.0)
	The presenter was easy to understand	3.50 (3.0–4.0)
	The volume of the video made it hard to hear	3.00 (1.0–4.0)
	*Total comprehensibility median score*	*3.22 (2.67*–*3.89)*
Credibility (0.747)	I could trust the information presented	3.50 (3.0–4.0)
	The person who presented this information seemed trustworthy	3.50 (2.0–4.0)
	I would prefer to get hepatitis C information from a different source	4.00 (3.0–4.0)
	The information was correct	3.50 (3.0–4.0)
	The information was complete	3.50 (2.0–4.0)
	*Total credibility median score*	*3.40 (2.8*–*4.0)*
Feasibility (0.703)	I listened carefully to the messages in the video	4.00 (3.0–4.0)
	The video gave me enough information to make a decision about hepatitis C screening	3.00 (2.0–4.0)
	A video like this could help me talk to my doctor about hepatitis C screening	3.50 (3.0–4.0)
	*Total feasibility median score*	*3.33 (3.0*–*4.0)*
Usability (0.397)	The video took too much time	3.00 (1.0–4.0)
	I am familiar with using a tablet device, such as an iPad	3.00 (2.0–4.0)
	I would need someone to help me use an iPad	3.00 (2.0–4.0)
	I would rather receive written information than view this information as a video on an iPad	3.00 (2.0–4.0)
	*Total usability median score*	*3.13 (2.5*–*3.8)*
Readability (N/A)	The words in the video were large enough to read	3.00 (3.0–4.0)
	I had problems seeing some of the graphics in the video because of the font size or color	3.00 (3.0–4.0)
	*Total readability median score*	*3.00 (3.0*–*4.0)*

Overall participants were positive about the patient-level intervention with one person saying, “Before I talked with you, I was much more frightened about the word Hep C.” Others showed appreciation for how much they learned; all of them indicated they felt prepared to talk to their doctor about HCV screening and would agree to be screened. When discussing areas for improvement, several participants indicated they would like information on why baby boomers are at a higher risk of infection. Furthermore, there was a sentence in the reminder letter that stated, “three out of four people infected with hepatitis C were born 1945–1965,” and most of the participants incorrectly interpreted that to mean three out of four baby boomers were infected with HCV. While participants said the information in the CDC pamphlet was helpful, and the CDC was described as credible, they generally qualified this statement by acknowledging that their opinion of CDC credibility was subject to change based on circumstance and political administration. They also indicated the video should be delivered with headphones or in a separate room from the waiting room, to avoid stigmatization. When asked who would be the best person to deliver the educational information (physician, epidemiologist), most patients indicated the credentials of the person did not matter, as long as they spoke with authority and gave accurate information.

#### Provider Intervention

In the survey, each part of the intervention (educational session, performance feedback e-mails) had a median score of at least 3 out of 4 on all constructs measured (Table 4). Provider feedback was further explained in the interviews. Most providers expressed interest in local data regarding HCV screening and prevalence since most of the data reported are national data.

**Table 4:** Provider-level intervention items and construct scores

EDUCATIONAL SESSION		
Construct (Cronbach’s alpha for reliability)	Items	Median score (range)
Acceptability (0.819)	I wanted more information from this session	3.00 (2.0–4.0)
	The educational session gave too much information	4.00 (2.0–4.0)
	I am going recommend hepatitis C virus screening to my eligible patients	4.00 (3.0–4.0)
	I will share the information I learned in the educational session with colleagues	4.00 (3.0–4.0)
	I would recommend this educational session to other providers	4.00 (3.0–4.0)
	This educational session should be made available to all primary care providers	4.00 (3.0–4.0)
	This educational session would be a waste of time for most primary care providers	4.00 (3.0–4.0)
	I can use the information from the educational session in my daily practice	4.00 (3.0–4.0)
	I don’t really need the information presented in the educational session	4.00 (3.0–4.0)
	*Total acceptability median score*	*3.78 (3.0–4.0)*
Comprehensibility (0.736)	It was easy to follow the information	4.00 (3.0–4.0)
	The information was clear	4.00 (3.0–4.0)
	The information flowed well	4.00 (3.0–4.0)
	This educational session taught me something new about hepatitis C screening	3.00 (3.0–4.0)
	I have changed my opinion about hepatitis C because of this information	3.00 (2.0–3.0)
	I know where to find helpful resources for hepatitis C screening	3.00 (2.0–4.0)
	The information was easy to understand	4.00 (3.0–4.0)
	The information was well organized	4.00 (3.0–4.0)
	*Total comprehensibility median score*	*3.63 (3.0–3.9)*
Credibility (0.823)	The information presented was credible	4.00 (3.0–4.0)
	The information presented was correct	4.00 (3.0–4.0)
	The information presented was up-to-date	4.00 (3.0–4.0)
	I would prefer to get hepatitis C information from a different source	3.00 (3.0–4.0)
	*Total credibility median score*	*3.75 (3.0–4.0)*
Feasibility (0.731)	The educational session took too much time	3.00 (2.0–4.0)
	I was satisfied with the information presented	4.00 (3.0–4.0)
	An educational session like this could help me talk to my patients about hepatitis C screening	4.00 (3.0–4.0)
	I need to learn more before I could strongly recommend hepatitis C virus screening to my patients	4.00 (1.0–4.0)
	*Total feasibility median score*	*3.50 (3.0–4.0)*
Usability (N/A)	I would rather receive written information, instead of a video	3.00 (2.0–4.0)
Readability (1.00)	I had problems seeing some of the graphics due to the colors and font used in the educational session	4.00 (3.0–4.0)
	The content was easy to read	4.00 (3.0–4.0)
	The appearance of the educational session was pleasant	4.00 (3.0–4.0)
	*Total readability median score*	*4.00 (3.0–4.0)*
FEEDBACK EMAILS		
Construct	Items	Median score (range)
Acceptability (N/A)	This information is important to providers	4.00 (3.0–4.0)
	I was satisfied with the information presented	4.00 (3.0–4.0)
	*Total acceptability median score*	*4.00 (3.0–4.0)*
Comprehensibility (N/A)	The content of the e-mail was easy to understand	4.00 (3.0–4.0)
Feasibility (N/A)	I would open and read the performance feedback e-mail every month	3.00 (3.0–4.0)
Usability (N/A)	I would prefer to get this information mailed to me, instead of e-mailed	4.00 (3.0–4.0)
Readability (N/A)	The graphics in the e-mail were clear	4.00 (3.0–4.0)

Providers noted the intervention would facilitate conversations with patients given limited time during a clinic visit with one noting the universal screening recommendation saves them from having to do an extensive risk assessment, saying, “But I’ve got 15 min, and there’s no way I'll ever get that down to that history, or have the time. So it’s just like: Oh, for God’s sakes, let me just screen you for Hep C.” Contrary to current guidelines, several providers did say they would assess risk of HCV infection by asking about the patient’s sexual history, even though sexual transmission of HCV is not common [21]. While most providers reported being aware that the guidelines for HCV screening were updated in March of 2020 to include adults ages 18–79, fewer were aware it is additionally recommended for all pregnant people.

Most providers indicated that they would prefer a primary care provider deliver the intervention. For the educational session format, one provider indicated in-person presentations typically have better engagement, but most of the other providers noted a virtual presentation would offer flexibility, especially for those working in rural clinics. Most providers preferred to receive the e-mails monthly, indicating they would also serve as a reminder to continue screening. Suggestions to improve the intervention included adding a systems-level intervention (e.g., standing orders). Overall, providers were positive about the intervention and indicated it was an appropriate amount of information and was comprehensive without being overly complicated or simplistic.

## Discussion

This reports the development and preliminary testing of a multilevel intervention to increase the uptake of HCV screening among baby boomers in a primary care setting. The patient-level intervention included a reminder letter, a CDC informational pamphlet, and a 7-min educational video. The provider-level intervention included an educational session and monthly performance feedback e-mails. The median for feasibility, acceptability, readability, and comprehensibility in all intervention items was above the a priori cutoff of ≥ 3. In qualitative interviews, both patients and providers expressed positive attitudes toward the intervention. Patients indicated they learned a considerable amount of information about HCV and felt ready to talk to their providers about getting screened. Providers indicated the education session material served as a knowledge refresher and would help them effectively communicate with patients given their restricted time during a patient visit.

We included a statement in our reminder letter that “three out of four people infected with hepatitis C were born 1945–1965.” However, most patients mistakenly interpreted this as, “three out of four baby boomers are infected with hepatitis C.” Health literacy and numeracy are considered critical factors to empower patients to take an active role in deciding their health care options [22]. Research shows patients with low numeracy have longer delays in seeking healthcare [23], which can increase the risk of poor health outcomes. Interventions that are designed with patient input allows researchers to develop content that is understandable to the population of interest, including patients with low health literacy and numeracy in order to maximize the effectiveness and improve health outcomes.

While most patients reported they believe the CDC is a credible source of health information, our participants indicated the reliability of the CDC depends on the current cultural and political climate. Furthermore, a recent study showed older adults (aged 26 +) had less trust in the CDC than younger adults, and only 64.6% adults reported trusting the CDC [24]. Our findings indicate interventions targeting baby boomers may need to refrain from including CDC or government-sponsored health information to be cognizant of the broader context of the current cultural and political zeitgeist.

Most providers indicated they would be more likely to attend an informational session if it was about reducing barriers to screening. Providers viewed the material as an opportunity to refresh their knowledge on information they already knew and as a chance to update their knowledge on new guidelines. Because the recommendation for universal screening for everyone aged 18–79 years was released in March 2020, much of the awareness was limited due to the fact that providers and healthcare facilities had limited time and resources at that time due to the COVID-19 pandemic [25]. Hospitals at that time shifted focus from providing preventive care and best outcomes for individual patients to adopting crisis standards of care to address the immediate needs of the population [25]. Therefore, new screening recommendations (e.g., for HCV screening) and cancer screening, in general, were lower priorities and did not receive as much attention as they would have under normal circumstances. Providers in our study reported experiencing competing demands on their time and tended to prioritize other cancer screenings over HCV screening during a limited clinic visit. They mentioned HCV screening was not incentivized and is not a quality indicator.

One notable finding in our study was that many providers seemed to focus on sexual transmission of HCV, even though that is not a primary mode of transmission [21]. While sexual transmission is possible, particularly among men who have sex with men and those who are co-infected with HIV, it is not how HCV is typically spread [21]. Currently, the most common way HCV is spread is blood-to-blood transmission through shared needles during injection drug use [21]. The method of infection for most baby boomer patients is unknown, but analyses suggest early HCV spread was a result of hospital-acquired infection as opposed to behavioral infection, suggesting infected baby boomers report no behavioral risk factors that would put them at a higher risk of infection [26]. Furthermore, 45% of people with HCV report no risk behaviors [6]. Therefore, it is important to ensure providers are aware of the most common patient risk factors so they can have effective conversations with them. The current guideline recommends universal screening, regardless of risk factors. Thus, providers should not be assessing risk factors, and this would further serve to reduce any potential stigma associated with a patient receiving HCV screening.

Our study has multiple strengths, including intervening on multiple levels, incorporating input from both providers and patients, and employing a mixed methods design. The results should be interpreted considering several limitations. First, while we achieved thematic saturation during our qualitative interviews, sample size, particularly for the surveys, was small, limiting our ability to detect meaningful differences in the responses. Second, participants self-selected into the study, which may have resulted in a sample that already had favorable attitudes towards HCV screening. Third, while our participants were racially and ethnically diverse, they were all from the same area in the Midwest and therefore may not be representative of other geographic locations. Lastly, due to the COVID-19 pandemic, we only delivered the intervention virtually via video conference and screen sharing. This may have affected the participants’ evaluation of the intervention. In addition, this limited our sample to only those who had internet and computer access, possibly removing participants of lower socioeconomic status or with lower digital literacy.

Although there has been a recommendation for universal screening of baby boomers for a decade, screening uptake remains low [4, 5]. This study developed and tested a multilevel intervention that was shown to be feasible, acceptable, and usable to patients and providers. After viewing the intervention materials, patients reported they felt prepared to talk to their providers about HCV screening. Providers felt the intervention materials would facilitate communication with their patients. Future research will modify the intervention for the expanded age group, assess intervention efficacy, and, if efficacious, disseminate broadly. This multilevel intervention has the potential to improve HCV screening in primary care and, ultimately, to reduce HCV-related morbidity and mortality.
