# Correction to: Development and Feasibility Testing of a Multilevel Intervention to Increase Hepatitis C Virus Screening Among Baby Boomers in Primary Care

**DOI:** 10.1007/s13187-023-02279-8

**Published:** 2023-03-10

**Authors:** Monica L. Kasting, Alfu Laily, Lauren D. Nephew, Cleveland G. Shields, Rivienne Shedd-Steele, Susan M. Rawl

**Affiliations:** 1 Department of Public Health, Purdue University, 812 W. State Street, Room 216, West Lafayette, IN 47907 USA; 2 Cancer Prevention and Control Program, Indiana University Simon Comprehensive Cancer Center, Indianapolis, IN USA; 3 Department of Medicine, Division of Gastroenterology and Hepatology, Indiana University School of Medicine, Indianapolis, IN USA; 4 Department of Human Development and Family Sciences, Purdue University, West Lafayette, IN USA; 5 Indiana University School of Nursing, Indianapolis, IN USA

**Correction to: Journal of Cancer Education**

10.1007/s13187-023-02268-x

The original version of this article unfortunately contained a mistake.

Table 3, p.8 of the article, the header REMINDER LETTER should be replaced with EDUCATIONAL VIDEO.

The original article has been corrected.

